# Supplementary material for: Exposure to Roundup and Antibiotics Alters Gut Microbial Communities, Growth, and Behavior in Rana berlandieri Tadpoles
Source: Biology (Basel). 2023 Aug 25;12(9):1171. doi: 10.3390/biology12091171 (PMC10525943; doi:10.3390/biology12091171)
Supplement: Supplementary file 1 [file biology-12-01171-s001.zip › biology-2551249-supplementary.pdf]

biology

MDPI

Supplementary Materials

Exposure to Roundup and Antibiotics Alters Gut Microbial Communities, Growth, and Behavior in *Rana berlandieri* Tadpoles

Table S1. Permuted community turn over between gut microbiome exposed to four treatments using Hill numbers.

Treatments		q = 0	q = 1	q = 2
Control vs. Roundup	Mean	47.52%	51.70%	62.99%
	Std. error	0.009	0.011	0.015
Control vs. Combination	Mean	68.44%	57.23%	58.91%
	Std. error	0.008	0.017	0.021
Control vs. Antibiotic	Mean	70.05%	60.04%	63.83%
	Std. error	0.007	0.012	0.015
Roundup vs. Combination	Mean	71.00%	66.18%	70.02%
	Std. error	0.008	0.014	0.017
Roundup vs. Antibiotic	Mean	72.87%	67.00%	71.53%
	Std. error	0.007	0.011	0.014
Combination vs. Antibiotic	Mean	65.17%	53.30%	52.81%
	Std. error	0.008	0.012	0.019

Table S2. Distinct microbial community members per treatment.

ASV	Genus	Coefficient	Q Value	P Value	Dunn Test	Treatments	P Value
ASV_103	*Streptococcus*	−0.10	3.42e−05	<0.0001	4.87	Antibiotic - Control	<0.0001
					4.23	Combination - Control	<0.0001
					4.96	Antibiotic - Roundup	<0.0001
					4.30	Combination - Roundup	<0.0001
ASV_2	*Niveispirillum*	−0.10	<0.0001	<0.0001	4.99	Antibiotic - Control	<0.0001
					4.49	Combination - Control	<0.0001
					4.70	Antibiotic - Roundup	<0.0001
					4.19	Combination - Roundup	<0.0001
ASV_1	NA	0.29	<0.0001	<0.0001	-3.21	Antibiotic - Control	<0.0001
					-3.05	Combination - Roundup	<0.0001
					2.22	Antibiotic - Roundup	<0.0001
					2.29	Combination - Roundup	<0.0001
					5.39	Control - Roundup	<0.0001
ASV_1174	*Microbacterium*	0.004	0.003	<0.0001	-4.19	Antibiotic - Control	<0.0001
					-4.12	Combination - Control	<0.0001
					2.90	Control - Roundup	0.007
ASV_8	*Azospirillum*	−0.07	0.027	<0.0001	4.15	Antibiotic - Control	<0.0001
					3.53	Combination - Control	<0.0001
					4.45	Antibiotic - Roundup	<0.0001
					3.82	Combination - Roundup	<0.0001
ASV_374	*Bacillus*	−0.03	0.03	<0.0001	3.54	Antibiotic - Control	0.002
					2.77	Combination - Control	0.01
					3.32	Antibiotic - Roundup	0.002
					2.53	Combination - Roundup	0.02
ASV_14	*Cetobacterium*	−0.17	0.04	<0.0001	3.60	Antibiotic - Control	0.002
					3.39	Combination - Control	0.002
					3.21	Antibiotic - Roundup	0.003
					3.00	Combination - Roundup	0.004
ASV_20	*Burkholderia-Caballeronia-Paraburkholderia*	−0.3	0.04	<0.0001	3.75	Antibiotic - Control	0.001
					3.34	Combination - Control	0.002
					3.05	Antibiotic - Roundup	0.005

ASV_395	*Polynucleobacter*	−0.04	0.04	<0.0001	2.65	Combination - Control	0.012
					3.26	Antibiotic - Control	0.001
					3.10	Antibiotic - Roundup	0.006
ASV_17	NA	0.09	0.05	<0.0001	-3.58	Antibiotic - Control	0.002
					-3.57	Combination - Control	0.001
					-2.56	Antibiotic - Roundup	0.02
					-2.56	Combination - Roundup	0.02
ASV_19	*Niveispirillum*	−0.02	0.05	0.0002	2.23	Antibiotic - Combination	0.05
					3.20	Antibiotic - Control	0.004
					3.64	Antibiotic - Roundup	0.002
ASV_231	*Galbitalea*	0.04	4.78e−06	<0.0001	-4.97	Antibiotic - Roundup	<0.0001
					-4.79	Combination - Roundup	<0.0001
					-3.57	Control - Roundup	<0.0001
ASV_103	*Streptococcus*	−0.09	3.38e−05	<0.0001	4.87	Antibiotic - Control	<0.0001
					4.23	Combination - Control	<0.0001
					4.96	Antibiotic - Roundup	<0.0001
					4.30	Combination - Roundup	<0.0001
ASV_402	NA	0.02	3.68e−05	<0.0001	-4.66	Antibiotic - Roundup	<0.0001
					-4.71	Combination - Roundup	<0.0001
					-3.58	Control - Roundup	<0.0001
ASV_10	*Bosea*	0.15	0.001	<0.0001	-3.87	Antibiotic - Roundup	0.0003
					-3.93	Control - Roundup	0.0005
					-3.33	Control - Roundup	0.001
ASV_160	*Bosea*	0.04	0.01	<0.0001	-2.99	Antibiotic - Control	0.004
					-3.06	Combination - Control	0.004
					-4.00	Antibiotic - Roundup	<0.0001
					-4.06	Combination - Roundup	<0.0001
ASV_9	*Xanthobacter*	0.16	0.01	<0.0001	-2.63	Antibiotic - Control	0.02
					-3.87	Antibiotic - Roundup	<0.0001
					-2.69	Combination - Roundup	0.02
ASV_687	NA	0.01	0.03	<0.0001	-3.95	Antibiotic - Control	<0.0001
					-2.96	Combination - Control	0.006
					-3.90	Antibiotic - Roundup	<0.0001
					-2.90	Combination - Roundup	0.006
